# Prolonged fibroblast growth factor 19 response in patients with primary sclerosing cholangitis after an oral chenodeoxycholic acid challenge

**DOI:** 10.1007/s12072-016-9769-7

**Published:** 2016-09-30

**Authors:** Serge J. Zweers, Elisabeth M. de Vries, Martin Lenicek, Dagmar Tolenaars, D. Rudi de Waart, Kiran V. K. Koelfat, Albert K. Groen, Steven W. M. Olde Damink, Ulrich Beuers, Cyriel Ponsioen, Peter L. M. Jansen, Frank G. Schaap

**Affiliations:** 1 Tytgat Institute for Liver and Intestinal Research, Amsterdam, The Netherlands; 2 Department of Gastroenterology and Hepatology, Academic Medical Center, Amsterdam, The Netherlands; 3 Department of Clinical Biochemistry and Laboratory Diagnostics, First Faculty of Medicine, Charles University in Prague, Prague, Czech Republic; 4 Department of Surgery, NUTRIM School for Nutrition and Translational Research in Metabolism, Maastricht University, PO BOX 616, 6200 MD Maastricht, The Netherlands; 5 Departments of Pediatrics, Laboratory Medicine, University of Groningen, University Medical Center Groningen, Groningen, The Netherlands

**Keywords:** Primary sclerosing cholangitis, Chenodeoxycholic acid, Fibroblast growth factor 19, Farnesoid X receptor

## Abstract

**Background:**

Bile salts likely contribute to liver injury in patients with primary sclerosing cholangitis (PSC) and primary biliary cholangitis (PBC). Fibroblast growth factor 19 (FGF19) is a bile salt-induced enterokine with hepatoprotective potential as it suppresses de novo bile salt synthesis. Here, we evaluated the bile salt receptor FXR/FGF19 gut–liver axis in PSC and PBC patients.

**Methods:**

Fasted patients with PSC (*n* = 12) and PBC (*n* = 10), and healthy controls (HC; *n* = 10) were orally challenged with the natural FXR agonist chenodeoxycholic acid (CDCA 15 mg/kg). Blood was sampled hourly until 8 h afterwards. Serum FGF19 and bile salt excursions were determined. Serum levels of 7α-hydroxy-4-cholesten-3-one (C4), reflecting bile salt synthesis, were measured as a biomarker of FGF19 response.

**Results:**

Baseline serum FGF19 levels were comparable between groups, while fasted bile salt levels in PSC patients were elevated. Upon CDCA challenge, HC and PBC patients showed a serum FGF19 peak after 4 h followed by a decline. PSC patients showed a prolonged and elevated serum FGF19 response up to 8 h, combined with a sustained serum elevation of CDCA and other bile salts. In general, C4 levels declined following FGF19 elevation. In PSC patients with less favorable prognosis, baseline C4 levels were drastically suppressed and did not further decline.

**Conclusion:**

Following an oral CDCA challenge, PSC patients showed an impaired clearance of CDCA and a prolonged serum FGF19 response. FXR agonist therapy in PSC could cause prolonged exposure to elevated levels of FGF19, and we propose careful monitoring for detrimental side effects in patient studies.

**Electronic supplementary material:**

The online version of this article (doi:10.1007/s12072-016-9769-7) contains supplementary material, which is available to authorized users.

## Introduction

Primary sclerosing cholangitis (PSC) is in its classical form a chronic cholestatic liver disease characterized by fibrotic strictures and saccular dilatations of the intra- and extrahepatic bile ducts [1]. Destruction and obliteration of medium- and large-sized bile ducts leads to bile retention, chronic cholestasis and biliary cirrhosis. The initial lesions in PSC are thought to be the consequence of a disturbed immunological response, with a possible role for bile or bile salts in perpetuating the disease [2].

Bile salt homeostasis in the liver is tightly regulated. Recent studies have indicated that bile salt synthesis in the liver is controlled by fibroblast growth factor 19 (FGF19) produced in the terminal ileum [3]. FGF19 is produced in ileocytes upon activation of the farnesoid X receptor (FXR) by bile salts absorbed from the intestinal lumen. Among the various bile salts, chenodeoxycholic acid (CDCA) is the most potent natural FXR-agonist [4]. Upon release into the portal circulation, FGF19 binds to FGFR4 at the sinusoidal membrane of hepatocytes. This leads to activation of MAP kinases and transcriptional repression of *CYP7A1*, encoding the rate-limiting enzyme in bile salt synthesis. In vivo, this repression can be followed by measuring serum levels of the bile salt intermediate 7α-hydroxy-4-cholesten-3-one (C4) [5]. By reducing bile salt synthesis, FGF19 has a hepatoprotective effect. A possible downside of FGF19 is its presumed tumorigenicity [6–8]. Prolonged exposure to high levels of FGF19 could contribute to the enhanced risk for bile duct, gallbladder and colon cancer in PSC [9–11].

The majority of patients with PSC have concomitant inflammatory bowel disease (IBD) and one may question if, in that setting, proper control of bile salt synthesis by the FXR/FGF19 axis is maintained. The objective of the present study was to evaluate this gut–liver signaling axis in patients with chronic cholestatic liver disease.

## Materials and methods

### Study subjects, oral CDCA challenge and blood sampling

Patients with PSC (*n* = 12) and primary biliary cholangitis (PBC, *n* = 10) were recruited from the outpatient hepatology clinic at the Academic Medical Center in Amsterdam. Healthy controls (HC, *n* = 10), matched for gender and age with the PSC patients, were approached via a written announcement.

The Mayo Risk Score (MRS) for PSC, a prognostic model used to estimate event-free survival and classified as low (MRS ≤ 0), intermediate (0 < MRS ≤ 2) or high (MRS > 2) risk, was calculated [12]. For the present study, MRS was re-categorized as low (MRS_low_; MRS ≤ 0, *n* = 7) and intermediate to high MRS (MRS_intermediate-high_; MRS > 0, *n* = 5).

The Mayo Risk Score (MRS) also has prognostic value for estimating event-free survival in PBC, and is classified as low (MRS ≤ 3.5), intermediate (3.5 < MRS ≤ 3.9) or high (MRS > 4) risk [13]. Note that calculation of MRS for PBC employs different parameters, yielding values that do not allow direct comparison with MRS for PSC.

PBC patients and those PSC patients taking ursodeoxycholic acid (UDCA) were asked to suspend UDCA use during 1 week before the start of the study. Following overnight fasting, all study subjects were orally administered chenodeoxycholic acid (CDCA; Sigma-Tau Arzneimittel, Dusseldorf, Germany) at a dose of 15 mg/kg body weight (total number of ingested 250 mg capsules was rounded downwards), based on a previous report of succesful pharmacologic activitation of FXR in human subjects [14].

Blood samples were obtained from an indwelling cannula placed in a cubital vein at baseline and at hourly intervals for up to 8 h after CDCA intake. During this sampling period, all participants refrained from food but had free access to water.

### Blood chemistry

Following collection of blood, serum was prepared and stored at −80 °C until analysis of FGF19, total bile salts, and 7α-hydroxy-4-cholesten-3-one (C4) levels at all sampling points. Alanine aminotransferase (ALT), gamma-glutamyltransferase (GGT) and alkaline phosphatase (ALP) were determined at baseline, 4 and 8 h after CDCA intake, using a Cobas Modular Analyzer (Roche Diagnostics, Germany).

FGF19 levels were determined by ELISA as previously described [15]. Total bile salt (TBS) levels were determined by an enzymatic cycling assay (Diazyme, Poway, CA, USA). Serum levels of C4 were determined by LC–MS as described in detail in the Supplemental Data. Serum bile salt composition was determined in samples collected at baseline and additional time points of interest, as described elsewhere [16].

### Statistical analysis

Differences between HC, PSC and PBC patient groups at baseline were evaluated using (non-)parametric ANOVA and post hoc tests with Bonferroni–Holm correction for multiple testing. Repeated measurement ANOVA was used to evaluate the response to CDCA in the respective study groups. In case of ALT, a non-parametric Friedman test for repeated measures was performed for each (sub)group. AUC_0-8h_ was calculated as net change from baseline using the trapezoid method. Statistical analyses were performed using SPSS v.20, and statistical significance was accepted at *p* < 0.05. Data in graphs are shown as median and interquartile range unless indicated otherwise, whereas data in tables are shown as median (range).

## Results

### Patient groups

Patient characteristics and liver biochemistry are shown in Table 1. In the PSC group, seven patients had a low MRS (MRS_low_), two patients had an intermediate MRS and three patients had a high MRS (grouped together as MRS_intermediate-high_). In the PBC group, three patients had a low MRS, two patients had an intermediate MRS and five patients had a high MRS. None of the study subjects had a history of bowel surgery. Of PSC patients, six had ulcerative colitis and two had Crohn’s disease, with a single patient per IBD subtype having mild terminal ileitis at the time of the study. UDCA was used by ten PSC patients (83 %), of which four temporarily discontinued its use. All PBC patients used UDCA, with a single patient refraining from discontinuation. The oral administration of CDCA with a median bolus of 1250 mg did not lead to side effects or complications in any of the subjects.

**Table 1:** Characteristics of the study subjects at baseline

	HC	PBC	PSC		
			All	MRS_low_	MRS_intermediate-high_
*n*	10	10	12	7	5
Male [*n* (%)]	5 (50)	0 (0)	7 (58)	3 (43)	4 (80)
Age (years) [median (range)]	51 (27–73)	63 (43–78)	48 (30–70)	46 (30–70)	50 (35–70)
Large duct PSC [*n* (%)]	N.A.	N.A.	12 (100)	7 (100)	5 (100)
Disease duration (years) [median (range)]	N.A.	10 (1–35)	11 (4–24)	7 (4–12)	17 (4–24)
Ulcerative colitis [*n* (%)]	0 (0)	0 (0)	6 (50)	3 (43)	3 (60)
Crohn’s disease [*n* (%)]	0 (0)	0 (0)	2 (17)	2 (29)	0 (0)
Ursodeoxycholic acid use [*n* (%)]	N.A.	10 (100)	10 (83)	6 (86)	4 (80)
AST (U/L) [median (range)] (*N*: < 40 U/L)	N.D.	29 (17–68)	53 (19–135)	38 (19–55)	105 (67–135)
ALP (U/L) [median (range)] (*N*: 40–120 U/L)	N.D.	107 (42–251)	272 (84–571)	214 (84–430)	366 (232–571)
Bilirubin total (µmol/L) [median (range)] (*N*: < 17 µmol/L)	N.D.	8 (5–17)	11 (5–85)	6 (5–12)	35 (20–85)
Albumin (g/L) [median (range)] (*N*: 35–50 g/L)	N.D.	42 (37–46)	40 (27–45)	43 (39–45)	33 (27–34)
Mayo Risk Score [median (range)]	N.A.	4.1 (2.9–5.9)	−0.2 (−1.3 to +2.4)	−0.8 (−1.3 to −0.1)	2.0 (+1.7 to +2.4)

Note that calculation of MRS for PBC employs different parameters, yielding values that do not allow direct comparison with MRS for PSC

*HC* healthy controls, *PBC* primary biliary cholangitis, *PSC* primary sclerosing cholangitis, *MRS* Mayo Risk Score, *AST* aspartate aminotransferase, *ALP* alkaline phosphatase, *N*.*A*. not applicable, *N*.*D*. not determined, *N*: normal range

### Serum bile salt levels and composition at baseline

**Fig. 1:**
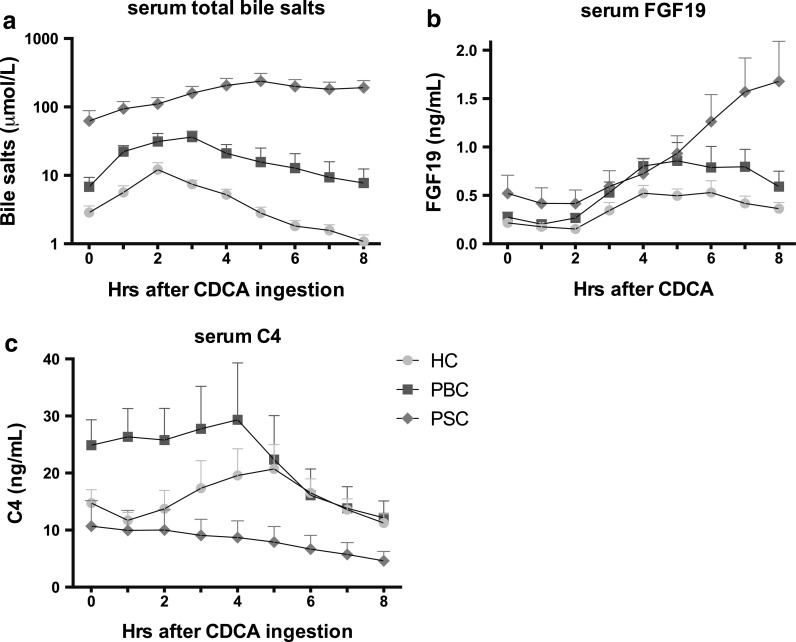
Serum bile salt, fibroblast growth factor 19 and C4 response curves following chenodeoxycholic acid (*CDCA*) administration. Serum was sampled hourly until 8 h after CDCA intake in healthy controls (*HC*, *green symbols*, *n* = 10), and patients with primary biliary cholangitis (*PBC*, *blue symbols*, *n* = 10) and primary sclerosing cholangitis (*PSC*, *red symbols*, *n* = 12). Data are expressed as mean values and standard error of the mean. *FGF19* fibroblast growth factor 19, *C4* 7α-hydroxy-4-cholesten-3-one

Total bile salt (TBS) levels at baseline were higher in the PSC than in the HC and PBC groups (Table 2; Fig. 1a). In PSC patients, TBS levels at baseline were considerably higher in the MRS_intermediate-high_ than in the MRS_low_ subgroup (Table 2; Suppl. Fig. 1A).

**Table 2:** Total bile salts, fibroblast growth factor 19 and C4 baseline values in healthy controls, PBC and PSC patients

	HC	PBC	PSC			*p* value			
			All	MRS_low_	MRS_intermediate-high_	HC vs PBC	HC vs PSC_All_	PSC_All_ vs PBC	MRS_low_ vs MRS_intermediate-high_
TBS *t* = 0 (μmol/L)	2.8 (0.1–6.8)	3.2 (1.2–26.9)	29.0 (6.0–329.3)	16.9 (6.0–13.8)	75.5 (67.4–329.3)	0.29	<0.001*	<0.001*	0.004*
FGF19 *t* = 0 (ng/mL)	0.18 (0.08–0.33)	0.23 (0.15–0.65)	0.26 (0.07–2.20)	0.24 (0.07–0.46)	0.58 (0.18–2.20)	0.20	0.15	0.72	0.06
C4 *t* = 0 (ng/mL)	17.1 (6.4–26.6)	18.6 (12.6–53.8)	5.9 (0.1–52.2)	10.9 (4.2–52.2)	0.6 (0.1–0.8)	0.11	0.048	0.005*	0.004*

Data are shown as median and range (minimum to maximum value)

Baseline TBS, FGF19 and C4 levels in HC were similar to previous reports [15, 33]

*HC* healthy controls, *PBC* primary biliary cholangitis, *PSC* primary sclerosing cholangitis, *MRS* Mayo Risk Score, *TBS* total bile salts, *FGF19* fibroblast growth factor 19, *C4* 7α-hydroxy-4-cholesten-3-one

* Statistical significance was accepted at *p* < 0.05 corrected for the number of comparisons made (post hoc Bonferroni–Holm correction)

**Fig. 2:**
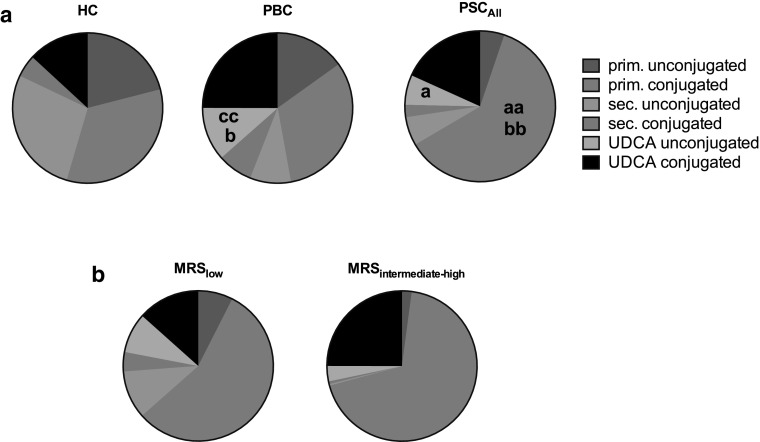
Serum bile salt composition at baseline. Serum bile salt composition was determined in healthy controls (HC, *n* = 3), PBC (*n* = 10) and PSC (*n* = 12) patients (**a**). Based on Mayo Risk Score (**b**), PSC patients were subcategorized as low risk (*n* = 7, MRS_low_) or moderate to high risk (*n* = 5, MRS_intermediate-high_). Bile salt composition is represented as (un)conjugated primary, secondary bile salts and UDCA. Data are expressed as mole fractions. The following* lowercase letters* denote differences of statistical significance: *a* and *aa* HC vs. PSC *p* < 0.05 and *p* < 0.005, respectively; *b* and *bb* PSC vs. PBC *p* < 0.05 and *p* < 0.005, respectively; *cc* HC vs. PBC *p* < 0.005. *HC* healthy controls, *PBC* primary biliary cholangitis, *PSC* primary sclerosing cholangitis, *UDCA* ursodeoxycholic acid

Serum bile salt composition at baseline is depicted in Fig. 2. The mole fraction of primary conjugated bile salts was higher in PSC than in PBC patients and HC (Fig. 2; Suppl. Table 3). Moreover, the mole fraction of unconjugated UDCA was elevated in PBC compared to PSC and HC, and elevated in PSC compared to HC (Fig. 2; Suppl. Table 3). Baseline elevation of serum bile salts in PSC was primarily due to increased levels of primary conjugated bile salts.

### Serum bile salt response to CDCA intake

Oral CDCA administration resulted in elevation of TBS in all groups (*p*_time_ < 0.001; Fig. 1a, Suppl. Fig. 1A). The serum bile salt response to CDCA was different between the three groups (*p*_time*group_ < 0.001), with the response in PSC patients deviating from that in HC and PBC patients (Suppl. Table 1). While a transient elevation of TBS was observed in HC and PBC patients, the response to CDCA in PSC patients showed an enhanced and prolonged elevation, which was predominantly due to a prolonged response in the PSC MRS_intermediate-high_ subgroup (Suppl. Figure 1; Suppl. Table 1).

The time-to-peak (TTP_TBS_) for TBS was similar in HC and PBC patients, but longer in PSC patients (Fig. 1; Suppl. Table 2). In 9 out of 12 PSC patients and 2 out of 10 PBC patients, a second peak in the individual serum response curves was observed between *T* = 5–8 h (data not shown). These findings are reflected in a higher AUC_TBS0-8h_ in PSC patients in comparison with HC and PBC patients (Suppl. Table 2). AUC_TBS0-8h_ values were similar in the MRS-based PSC subgroups (Suppl. Table 2).

**Fig. 3:**
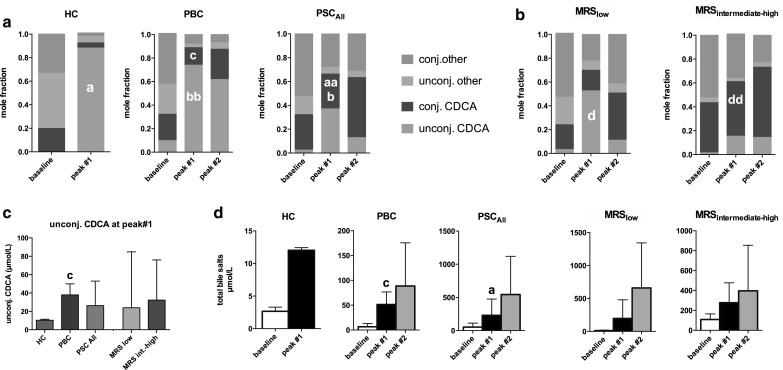
Serum bile salt composition and peak levels following CDCA administration. Bile salt composition was determined in samples from healthy controls (HC, *n* = 3), PBC (*n* = 10) and PSC (*n* = 12) patients at baseline and at time of peak(s) of total bile salts. Data are expressed as mole fractions and categorized as (un)conjugated CDCA and other bile salts (**a**). Based on Mayo Risk Score (**b**), PSC patients were subcategorized as low (*n* = 7, MRS_low_) or intermediate to high risk (*n* = 5, MRS_intermediate-high_). Unconjugated CDCA absolute concentration was measured at first total bile salt peak (**c**) in the same (sub)groups used for (**a**) and (**b**). For the same groups, total bile salt peak(s) are shown as absolute concentrations (**d**). For peak#2: PBC (*n* = 2), PSC_All_ (*n* = 9), PSC MRS_low_ (*n* = 5) and PSC MRS_intermediate-high_ (*n* = 4). Data in (**c**) and (**d**) are expressed as median and interquartile range. The following *lowercase letters* denote differences of statistical significance: *a* and *aa* HC vs. PSC *p* < 0.05 and *p* < 0.005, respectively; *b* and *bb* PSC vs. PBC *p* < 0.05 and *p* < 0.005, respectively; *c* HC vs. PBC *p* < 0.05; *d* and *dd* PSC_low_ vs. PSC_moderate-high_ *p* < 0.05 and *p* < 0.005, respectively. *HC* healthy controls, *PBC* primary biliary cholangitis, *PSC* primary sclerosing cholangitis, *CDCA* chenodeoxycholic acid

For each of the subjects, serum bile salt pool composition was analyzed at the individual peak level, and peak TBS level was calculated from the sum of individual bile salt species (Fig. 3a). At the first peak, TBS levels were higher in PSC and PBC patients than in HC (Fig. 3d), whereas unconjugated CDCA was higher in PBC patients than in HC (Fig. 3c). Peak mole fraction of conjugated CDCA was highest in PSC (Fig. 3a). Moreover, MRS_intermediate-high_ PSC patients had increased conjugated CDCA levels compared to MRS_low_ PSC patients (Fig. 3b). No differences were found between bile salt levels or composition at the time of the second peak, when comparing PSC with PBC patients or MRS_low_ PSC patients with MRS_intermediate-high_ PSC patients (Fig. 3a, b, d).

### FGF19 levels at baseline and serum response after CDCA

Baseline serum FGF19 levels in both patient groups and controls were comparable (Fig. 1b; Table 2). CDCA caused a gradual increase in serum FGF19 levels in all groups (*p*_time_ < 0.001; Fig. 1b, Suppl. Fig. 1A). The serum FGF19 response to CDCA was different between groups (*p*_time*group_ = 0.018), with the response in PSC patients deviating from that in HC (Suppl. Table 1). The first half of the FGF19 response curves was similar in the three groups. During the second half, the FGF19 response showed a persistent elevation in PSC patients in contrast to a decline in PBC patients and HC (Fig. 1b, Supp. Table 2).

### C4 levels at baseline and serum response after CDCA

In PSC patients, C4 levels at baseline were decreased (Fig. 1c; Table 2). This is attributable to the MRS_intermediate-high_ subgroup where C4 levels reached the lower limit of quantification (i.e. 0.1 ng/mL). Levels of C4 in the MRS_low_ subgroup were not different from those in HC or PBC patients (Supp. Fig. 1C; Table 2).

The serum C4 response to oral CDCA administration was significant in HC (*p* = 0.018), in PBC patients (*p* < 0.001) and in the MRS_low_ subgroup (*p* = 0.006), but not in the MRS_intermediate-high_ subgroup (*p* = 0.64). HC and PBC patients showed a decrease in C4 levels in the second half of the response curve, following the peak of FGF19 levels at 4–5 h (Fig. 1c; Suppl. Table 2). A similar reduction of C4 levels preceded by elevation of serum FGF19 was noted in the MRS_low_ subgroup (Suppl. Fig. 1C). Serum C4 levels in the MRS_intermediate-high_ PSC subgroup were already low at baseline, and CDCA ingestion had no further effect (Suppl. Fig. 1C).

## Discussion

In this study, we evaluated the gut–liver axis of FGF19-mediated regulation of bile salt synthesis in PSC and PBC patients by the oral administration of the natural FXR agonist CDCA. In PBC patients and in healthy controls, serum FGF19 levels peaked 4 h after oral CDCA and then declined. In contrast, the serum FGF19 response curve in PSC patients showed a prolonged response, with elevated serum FGF19 levels up to 8 h after oral CDCA administration. What could be the mechanism behind this prolonged FGF19 response in PSC patients?

CDCA is the most potent FXR agonist among the natural bile salts [4]. Passive or carrier-mediated absorption of bile salts in the terminal ileum results in FXR activation and *FGF19* induction. FGF19 circulates from the ileum to the liver where it suppresses bile salt synthesis [17]. While FGF19 is not expressed in normal human hepatocytes, under cholestatic conditions, human liver produces FGF19 [18]. Whether this occurs in hepatocytes or cholangiocytes is not clear. A previous report showed a correlation between serum and liver levels of FGF19 protein and severity of cholestasis in PBC [19]. Although the regulation of *FGF19* in the liver is not entirely understood, it is likely that FGF19 synthesis in the cholestatic liver is under the control of FXR as it is in the ileum and can hence be induced by CDCA.

Our study showed a prolonged serum FGF19 response in PSC patients after oral administration of CDCA. The TBS and FGF19 response curves showed that, in all groups, TBS elevation preceded serum FGF19 elevation. In PSC patients, persistent elevation of TBS was followed by elevated FGF19 until 8 h after oral intake of CDCA, suggesting that the decreased clearance of CDCA and other bile salts in these patients results in prolonged FGF19 production. While FGF19 during the initial phase of the response curve most likely derives from the terminal ileum due to FXR activation by luminally absorbed CDCA, we speculate that FGF19 in the second phase of the response curve may originate from both the liver and ileum. Prolonged elevation of serum CDCA could stimulate FGF19 production in ileal enterocytes via basolateral diffusion of unconjugated CDCA or via absorption and stimulation of FXR in hepatocytes or cholangiocytes. The difference in response between PSC and PBC patients may be related to the degree of cholestasis, since PSC patients had higher levels of ALP and TBS than PBC patients (Tables 1, 2; Fig. 1a). However, we did not observe a correlation between cholestatic parameters and FGF19 levels (data not shown). In addition, FGF19 continued to rise until 8 h after oral administration of CDCA in both MRS_low_ and cholestatic MRS_intermediate-high_ PSC patients (Suppl. Fig. 1B). To further study the influence of cholestasis on the serum FGF19 response following an oral CDCA challenge, it could be informative to evaluate this response in patients with severe cholestasis, e.g., in patients with malignant bile duct obstruction. We alternatively considered the possibility that the difference in FGF19 response between PSC and PBC patients may be disease-specific, rather than related to differences in severity of cholestasis. In PSC, intermediate and large bile ducts contain numerous strictures. Proximal to these stenoses, the biliary pressure is likely elevated, leading to the characteristic saccular dilatations. Bile duct damage is different in PBC where only the small intrahepatic bile duct(ule)s close to the site of hepatocellular secretion are affected. Albeit difficult to prove, it is possible that in PSC bile salts leak from these injured bile ducts, especially from pre-stenotic segments that may be under increased biliary pressure. This leakage could be enhanced upon an increase in biliary pressure after canalicular secretion of administered CDCA. As a result, bile salts recirculating to the liver via such speculated cholehepatic shunting could cause prolonged hepatic FXR activation and consequential hepatic FGF19 synthesis. Moreover, FGF19 from bile could leak into the circulation and directly contribute to elevated serum levels of FGF19 after CDCA challenge in PSC [20]. The latter scenario is less likely, as cholestasis and liver injury markers were not affected by CDCA ingestion (Suppl. Fig. 2).

Following a CDCA challenge, a larger fraction of CDCA was conjugated in PSC patients at peak bile salt level than in PBC patients. This especially occurred in the MRS_intermediate-high_ PSC subgroup (Fig. 3a, b). Also at baseline, PSC patients showed increased primary conjugated bile salts, which largely accounted for elevated TBS (Fig. 2). Since the liver is the only site where bile salt conjugation occurs, conjugated bile salts in serum must originate from the liver. Therefore, conjugated CDCA in serum either originates from leaky bile ducts or from reversed basolateral efflux from hepatocytes. In the absence of a stimulus for gallbladder contraction, intestinal reabsorption following enterohepatic circulation seems a less likely explanation for serum appearance of conjugated CDCA.

UDCA is a very weak FXR agonist [4]. In order to prevent potential interference with CDCA effects, all participants were requested to discontinue use of UDCA for 1 week. Longer discontinuation may have been preferable for our study, but was not justified from an ethical and medical point of view. UDCA was not discontinued by all subjects. As a result, unconjugated UDCA remained elevated in PSC and PBC patients (Fig. 2). However, there was no correlation between baseline UDCA levels and FGF19 or C4 levels (data not shown).

At baseline, PSC patients in the MRS_intermediate-high_ group had very low levels of C4 and normal levels of FGF19 (Suppl. Fig. 1C). This could point to strongly suppressed bile salt synthesis via FGF19-independent pathways in progressive stages of the disease. Strongly suppressed synthesis of (primary) bile salts can be expected to result in a greater proportion of gut flora-derived secondary bile salts in the circulating pool. Yet, primary bile salts comprise the main bile salt species in PSC. It will be interesting to learn if *CYP7A1*-independent routes of bile salt synthesis, which do not result in the formation of the bile salt intermediate C4, are operating in PSC.

The findings of our study might be relevant for FXR agonist-based therapy. A putative beneficial effect of FXR agonists in PSC patients would be the suppression of bile salt synthesis via FGF19 induction. Our data suggest that PSC patients with intermediate–high MRS may not benefit from this therapy, given the fact that bile salt synthesis, at least via the classical pathway, appears to be already low. However, FXR agonists may still have beneficial effects on properties of the bile salt pool as well as gut barrier function, exert anti-inflammatory action in intestine and liver, and promote repair of cholestatic injury [21–26].

Our finding of prolonged FGF19 elevation in PSC patients following CDCA intake may have additional implications for FXR agonist-based therapy in this disease. Obeticholic acid (OCA) is an approximately 100-fold more potent FXR agonist than CDCA and is under current investigation in a phase 2 trial in PSC patients [27, 28]. In PBC patients, OCA at a dose of 10 mg was observed to result in a ca. twofold elevation of serum FGF19 [29]. This approximates the effect of CDCA in PBC patients in our current study, as well as postprandial FGF19 changes in healthy subjects [15, 30]. Based on our findings, prolonged elevation of FGF19 may be anticipated in PSC patients using OCA. Prolonged exposure to elevated FGF19 levels may theoretically increase the risk of PSC patients for developing cholangiocarcinoma and other malignancies, as has been shown in mice [31].

There are some limitations to our study. As the physiological stimulus of an oral fat load did not result in a uniform serum FGF19 response (unpublished results), we chose oral CDCA to induce intestinal FGF19 release. The orally administered CDCA was not conjugated and is expected to be taken up in part by the proximal small intestine (which has little *FXR* and no *FGF19* expression) via passive diffusion [32]. Thus, oral CDCA ingestion does not reflect normal physiology in which conjugated bile salts are delivered to the terminal ileum by a meal-stimulated gallbladder contraction. In addition, the PBC patients had lower cholestatic parameters than the PSC patients, and as such may be a suboptimal control group for cholestatic disease. Moreover, to test our hypothesis of CDCA-induced hepatic production of FGF19 in PSC, analysis of liver biopsies taken before and after CDCA would be needed. However, such invasive steps are ethically not allowed.

Taken together, we demonstrate that PSC patients show a prolonged FGF19 response after an oral dose of CDCA. FXR-agonist therapy in PSC can cause prolonged exposure to elevated levels of FGF19. Its previously reported hepatoproliferative properties warrant evaluation of the long-term effects of elevated FGF19 levels in PSC patients.

## Electronic supplementary material

Below is the link to the electronic supplementary material.

Supplementary material 1 (DOCX 717 kb)
