# Supplementary material for: Prolonged fibroblast growth factor 19 response in patients with primary sclerosing cholangitis after an oral chenodeoxycholic acid challenge
Source: Hepatol Int. 2016 Sep 30;11(1):132–40. doi: 10.1007/s12072-016-9769-7 (PMC5233735; doi:10.1007/s12072-016-9769-7)
Supplement: Supplementary file 1 — Supplementary material 1 (DOCX 717 kb) [file 12072_2016_9769_MOESM1_ESM.docx]

**Supplemental Data**

Analysis of C4 levels

*Materials*

LC-MS grade methanol (Biosolve BV, The Netherlands), LC-MS grade ammonium acetate (Sigma-Aldrich, USA).

*Method*

One hundred microliters of serum and 2 ng of deuterium labelled internal standard (7α-hydroxy-4-cholestene-3-one d7, Santa Cruz Biotechnology, USA) in 40 µL of methanol were mixed and extracted as previously reported^1^. Purified sample was dissolved in 50 µL of 75% methanol, 15 µL were injected on HPLC system (Dionex Ultimate 3000, Dionex Softron GmbH, Germany) equipped with Hypersil GOLD column (150x2.1 mm, 3 µm, Thermo Scientific, USA) and Security Guard column (Phenomenex, USA). Sample was eluted with methanol:water:ammonium acetate (flow rate 0.3 mL/min) at 40°C. While ammonium acetate concentration was kept at 0.1% (w/v) at all times, methanol concentrations (v/v) were as follows: 1.-8. min 82 %-90 %; 8.-10. min 90 %; 10.-12. min 99 %; 12.-17. min 82 %.

Triple quadrupole mass spectrometer (TSQ Quantum Access Max with H-ESI II probe, Thermo Fisher Scientific, Inc., USA) operating in SRM mode served as detector. Transitions used for monitoring of C4 and internal standard were: m/z 401.4 → 177.3, 401.4 → 383.6 and 408.4 → 184.3, 408.4 → 390.6, respectively.

1. Lenicek M, Juklova M, Zelenka J et al. Improved HPLC Analysis of Serum 7{alpha}-Hydroxycholest-4-en-3-one, a Marker of Bile Acid Malabsorption. Clin Chem. 2008;54:1087-88.

**Supp. Figure 1.** **Serum bile salt, fibroblast growth factor 19 and C4 response curves following CDCA administration.** Serum was sampled hourly until 8 hours after CDCA intake in healthy controls (HC, green symbols, n=10), PBC patients (blue symbols, n=10) and PSC patients with low or intermediate-high Mayo Risk Score (MRS_low_, orange symbols, n=7 and MRS_intermediate-high,_ purple symbols, n=5, resp.). Data are expressed as mean values and standard error of the mean.

FGF19, Fibroblast Growth Factor 19; C4, 7α-hydroxy-4-cholesten-3-one; CDCA, chenodeoxycholic acid; PBC, primary biliary cholangitis; PSC, primary sclerosing cholangitis.

**Supp. Figure 2. Liver enzyme tests after CDCA intake.**

Alanine aminotransferase, gamma-GT and alkaline phosphatase were determined in serum sampled 0, 4 and 8 hours after CDCA intake in healthy controls (HC, green symbols, n=5), and patients with PBC (blue symbols, n=10) and PSC (red symbols, n=12). Data are shown as median values and interquartile range.

ALT, alanine aminotransferase; GGT, gamma-GT; ALP, alkaline phosphatase; CDCA, chenodeoxycholic acid; PBC, primary biliary cholangitis; PSC, primary sclerosing cholangitis; n.s., not significant.

**Supp. Table 1. Differences in trend of response curves of total bile salts, Fibroblast Growth Factor 19 and C4 in healthy controls, PBC and PSC patients**

|  | **P value** | | | |
| --- | --- | --- | --- | --- |
|  | **HC**  **vs**  **PBC** | **HC**  **vs**  **PSC_All_** | **PSC_All_**  **vs**  **PBC** | **MRS_Low_**  **vs**  **MRS_Int.-high_** |
| **TBS curve** | **0.022*** | **<0.001*** | **<0.001*** | **0.023*** |
| **FGF19 curve** | **0.026** | **0.004*** | **1.0** | **0.77** |
| **C4 curve** | **1.0** | **0.007*** | **0.004*** | **<0.001*** |

Repeated measures ANOVA with post-hoc testing was performed.

Statistical significance was accepted at P<0.05 corrected for the number of comparisons made (post-hoc Bonferroni-Holm correction) and is demarcated by * in the table.

HC, healthy controls; PBC, primary biliary cholangitis; PSC, primary sclerosing cholangitis; MRS, Mayo Risk Score; TBS, total bile salts; FGF19, Fibroblast Growth Factor 19; C4, 7α-hydroxy-4-cholesten-3-one.

|  | **HC** | **PBC** | **PSC** | | | **P value** | | | |
| --- | --- | --- | --- | --- | --- | --- | --- | --- | --- |
|  |  |  | **All** | **MRS**  **_low_** | **MRS**  **_int.-high_** | **HC**  **vs**  **PBC** | **HC**  **vs PSC_All_** | **PSC_All_**  **vs**  **PBC** | **MRS_low_**  **vs**  **MRS_int.-high_** |
| **AUC TBS**  (μmol·L^-1^·h^-1^) | **17.04**  **[-24.6-39.1]** | **87.87**  **[-33.6-400.5]** | **335.3**  **[-11.7-2589.0]** | **173.2**  **[17.5-2589]** | **786.1**  **[-11.7-2339.0]** | **0.037** | **<0.001*** | **0.005*** | **0.204** |
| **AUC FGF19**  (ng·mL^-1^·h^-1^) | **0.88**  **[-0.15-2.83]** | **1.93 [0.02-6.82]** | **1.88**  **[-2.24-14.61]** | **3.62 [0.95-14.61]** | **1.23**  **[-2.24-1.85]** | **0.028** | **0.019** | **1.00** | **0.042** |
| **AUC C4**  (ng·mL^-1^·h^-1^) | **1.48**  **[-39.0-53.4]** | **-40.8**  **[-89.5-230.1]** | **-1.09**  **[-221.1-54.42]** | **-15.6**  **[-221.1-54.4]** | **1.25**  **[-2.1-4.45]** | **0.658** | **0.264** | **0.014*** | **<0.001*** |
| **TTP TBS**  (hrs) | **2.4**  **[0.0-**  **4.0]** | **2.5**  **[1.0-4.0]** | **4.5**  **[1.0-**  **8.0]** | **5.0**  **[1.0-6.0]** | **5.0**  **[4.0-**  **8.0]** | **1.00** | **0.015** | **0.022** | **0.152** |
| **TTP FGF19**  (hrs) | **4.5**  **[3.0-**  **8.0]** | **4.5**  **[3.0-7.0]** | **8.0**  **[4.0-**  **8.0]** | **8.0**  **[7.0-8.0]** | **8.0**  **[4.0-**  **8.0]** | **0.753** | **0.002*** | **<0.001*** | **0.56** |
| **TTP C4**  (hrs) | **3.8**  **[0.0-8.0]** | **1.5**  **[0.0-4.0]** | **2.8**  **[0.0-**  **6.0]** | **3.0**  **[0.0-6.0]** | **3.0**  **[0.0-**  **6.0]** | **0.025** | **0.32** | **0.16** | **0.93** |
| **TBS Peak#1**  (μmol/L)  (n=) | **9.6**  **[8.3-**  **10.1]**  **(n=10)** | **40.8**  **[21.7-83.7]**  **(n=10)** | **51.9**  **[9.5-740.2]**  **(n=12)** | **49.9**  **[5.0-740.2]**  **(n=7)** | **127.7**  **[22.9-516.1]**  **(n=5)** | **0.011*** | **0.03** | **0.39** | **0.69** |
| **TBS Peak#2**  (μmol/L)  (n=) | **N.A.** | **76.1**  **[23.6-128.6]**  **(n=2)** | **170.2**  **[5.4-1593.4]**  **(n=9)** | **584.5**  **[5.4-1593.4]**  **(n=4)** | **170.2**  **[72.9-964.7]**  **(n=5)** | **N.A.** | **N.A.** | **0.35** | **1.0** |
| **FGF19 t=4**  (ng/mL) | **0.47 [0.13-0.79]** | **0.93 [0.51-1.21]** | **0.51 [0.13-1.82]** | **0.55 [0.13-1.12]** | **0.48 [0.18-1.82]** | **0.007*** | **0.47** | **0.15** | **0.57** |
| **FGF19 t=8**  (ng/mL) | **0.30 [0.12-0.71]** | **0.46 [0.21-2.10]** | **0.96 [0.44-4.87]** | **0.97 [0.88-4.87]** | **0.84 [0.44-2.52]** | **0.07** | **<0.001*** | **0.004*** | **0.17** |
| **FGF19 peak**  (ng/mL) | **0.64**  **[0.28-1.33]** | **1.24**  **[0.81-2.52]** | **1.13**  **[0.81-4.87]** | **0.97**  **[0.60-2.86]** | **1.01**  **[0.90-4.87]** | **0.016*** | **<0.001*** | **0.22** | **0.57** |
| **C4 peak**  (ng/mL) | **7.0**  **[0.0-23.6]** | **2.45**  **[0.0-77.2]** | **1.6**  **[0.0-15.9]** | **3.8**  **[0.0-15.9]** | **0.4**  **[0.0-**  **2.1]** | **0.40** | **0.12** | **0.79** | **0.16** |

**Supp. Table 2. Parameters from total bile salts, Fibroblast Growth Factor 19 and C4 curves after chenodeoxycholic acid intake in healthy controls, PBC and PSC patients**

Data are shown as median and range [minimum to maximum value]. Statistical significance was accepted at P<0.05 corrected for the number of comparisons made (post-hoc Bonferroni-Holm correction) and is demarcated by * in the table.

AUC, area under the curve; TTP, time to peak; HC, healthy controls; PBC, primary biliary cholangitis; PSC, primary sclerosing cholangitis; MRS, Mayo Risk Score; TBS, total bile salts; FGF19, Fibroblast Growth Factor 19; C4, 7α-hydroxy-4-cholesten-3-one.

**Supp. Table 3. Serum bile salt composition and bile salt levels at baseline and at first and second bile salt peak**

|  | **HC** | **PBC** | **PSC** | | | **P value** | | | |
| --- | --- | --- | --- | --- | --- | --- | --- | --- | --- |
|  |  |  | **All** | **MRS**  **_low_** | **MRS**  **_int.-high_** | **HC**  **vs**  **PBC** | **HC vs PSC_All_** | **PSC_All_**  **vs**  **PBC** | **MRS_low_**  **vs**  **MRS_int.-high_** |
| **CDCA Unconj.**  **at t=0**  (μmol/L) | **0.0**  **[0.0-2.6]** | **0.32**  **[0.01-3.1]** | **0.0**  **[0.0-12.9]** | **0.0**  **[0.0-**  **1.3]** | **0.0**  **[0.0-12.9]** | **0.012*** | **0.86** | **0.005*** | **0.34** |
| **UDCA Unconj.**  **at t=0**  (μmol/L) | **0.0**  **[0.0-0.0]** | **0.087**  **[0.03-0.26]** | **0.005**  **[0.0-0.33]** | **0.0**  **[0.0-0.33]** | **0.01**  **[0.0-014]** | **<0.001*** | **0.012*** | **0.024*** | **0.93** |
| **Prim. Unconj.**  **at t=0**  (mole fract.)  (n=) | **0.15 [0.0-0.80**  **(n=10)** | **0.16 [0.01-0.29]**  **(n=10)** | **0.00 [0.00-0.46]**  **(n=12)** | **0.00 [0.00-0.46]**  **(n=7)** | **0.01 [0.00-0.08]**  **(n=5)** | **0.73** | **0.06** | **0.003*** | **0.93** |
| **Prim. Conj.**  **at t=0**  (mole fract.) | **0.26 [0.15-0.63]** | **0.38 [0.10-0.51]** | **0.61 [0.28-0.92]** | **0.56 [0.28-0.77** | **0.72 [0.48-0.92]** | **0.82** | **0.005*** | **<0.001*** | **0.29** |
| **Sec. Unconj.**  **at t=0**  (mole fract.) | **0.32 [0.00-0.71]** | **0.07 [0.00-0.24]** | **0.01 [0.00-0.27]** | **0.07 [0.00-0.27]** | **0.00 [0.00-0.01]** | **0.31** | **0.09** | **0.17** | **0.12** |
| **Sec. Conj.**  **at t=0**  (mole fract.) | **0.00 [0.00-0.17]** | **0.08 [0.00-0.16]** | **0.01 [0.00-0.10]** | **0.03 [0.00-0.10]** | **0.01 [0.00-0.02]** | **0.16** | **0.84** | **0.024** | **0.12** |
| **UDCA Unconj.**  **at t=0**  (mole fract.) | **0.00 [0.00-0.00]** | **0.09 [0.03-0.26]** | **0.01 [0.00-0.33]** | **0.00 [0.00-0.33]** | **0.01 [0.00-0.14]** | **<0.001*** | **0.012*** | **0.024*** | **0.93** |
| **UDCA Conj.**  **at t=0**  (mole fract.) | **0.08 [0.03-0.34]** | **0.24 [0.00-0.60]** | **0.19 [0.00-0.50]** | **0.13 [0.00-0.28]** | **0.24 [0.08-0.50]** | **0.26** | **0.60** | **0.58** | **0.22** |
| **Prim. Total**  **at t=0**  (mole fract.) | **0.57 [0.19-0.95]** | **0.57 [0.15-0.66]** | **0.66 [0.48-0.92]** | **0.62 [0.52-0.77]** | **0.72 [0.48-0.92]** | **0.50** | **0.08** | **0.010*** | **0.47** |
| **UDCA Total**  **at t=0**  (mole fract.) | **0.08 [0.03-0.34]** | **0.31 [0.05-0.79]** | **0.27 [0.00-0.51]** | **0.27 [0.00-0.37]** | **0.27 [0.08-0.51]** | **0.03** | **0.11** | **0.51** | **0.68** |
| **Conj. Total**  **at t=0**  (mole fract.) | **0.38 [0.20-0.97]** | **0.68 [0.37-0.76]** | **0.89 [0.28-1.00]** | **0.77 [0.28-1.00]** | **0.99 [0.77-1.00]** | **0.29** | **0.015*** | **0.010*** | **0.07** |
| **CDCA Unconj.**  **at Peak#1**  (μmol/L) | **10.3**  **[10.2-11.3]** | **38.0**  **[14.0-61.2]** | **26.3**  **[0.0-259.7]** | **35.6**  **[8.2-259.7]** | **23.6**  **[0.0-54.8]** | **0.011*** | **0.19** | **0.47** | **0.37** |
| **CDCA Unconj.**  **at Peak#1**  (mole fract.)  (n=) | **0.87 [0.82-0.96]**  **(n=3)** | **0.78 [0.54-0.89]**  **(n=10)** | **0.36 [0.0-0.83]**  **(n=12)** | **0.53 [0.11-0.83]**  **(n=7)** | **0.11 [0.0-0.32]**  **(n=5)** | **0.18** | **0.014*** | **0.001*** | **0.012*** |
| **CDCA Conj.**  **at Peak #1**  (mole fract.) | **0.03 [0.03-0.07]** | **0.13 [0.07-0.37]** | **0.22 [0.11-0.83]** | **0.14 [0.11-0.33]** | **0.44 [0.26-0.63]** | **0.011*** | **0.009*** | **0.027*** | **0.007*** |
| **Other Unconj.**  **at Peak#1**  (mole fract.) | **0.08 [0.0-0.08]** | **0.02 [0.0-0.09]** | **0.03 [0.0-0.25]** | **0.06 [0.0-0.25]** | **0.003**  **[0.0-0.12]** | **0.61** | **0.83** | **1.0** | **0.16** |
| **Other Conj.**  **at Peak #1**  (mole fract.) | **0.02 [0.01-0.03]** | **0.05 [0.01-0.24]** | **0.25 [0.01-0.65]** | **0.15 [0.01-0.61]** | **0.26 [0.12-0.65]** | **0.09** | **0.043** | **0.025** | **0.37** |
| **Total Conj.**  **at Peak #1**  (mole fract.) | **0.05 [0.04-0.10]** | **0.18 [0.10-0.45]** | **0.62 [0.13-1.00]** | **0.35 [0.13-0.78]** | **0.80 [0.68-1.00]** | **0.011*** | **0.009*** | **0.006*** | **0.012*** |
| **Total bile salts**  **at Peak#1**  (μmol/L) | **11.8 [11.8-12.5]** | **44.5 [25.0-105.3]** | **139.2 [12.4-763.3]** | **58.1 [12.4-763.3]** | **227.2 [74.6-609.9]** | **0.011*** | **0.014*** | **0.048** | **0.17** |
| **CDCA Unconj.**  **at Peak# 2**  (mole fract.)  (n=) | **N.A.** | **0.62 [0.39-0.84]**  **(n=2)** | **0.12 [0.00-0.32]**  **(n=9)** | **0.06 [0.00-0.32]**  **(n=4)** | **0.14 [0.02-0.26]**  **(n=5)** | **N.A.** | **N.A.** | **0.034** | **0.46** |
| **CDCA Conj.**  **at Peak #2**  (mole fract.) | **N.A.** | **0.26 [0.12-0.40]** | **0.51 [0.20-0.79]** | **0.41 [0.20-0.58]** | **0.58 [0.30-0.79]** | **N.A.** | **N.A.** | **0.16** | **0.14** |
| **Other Unconj.**  **at Peak#2**  (mole fract.) | **N.A.** | **0.06 [0.01-0.10]** | **0.002 [0.00-0.31]** | **0.002 [0.00-0.31]** | **0.002 [0.00-0.19]** | **N.A.** | **N.A.** | **0.23** | **0.80** |
| **Other Conj.**  **at Peak #2**  (mole fract.) | **N.A.** | **0.07 [0.03-0.11]** | **0.25 [0.10-0.69]** | **0.43 [0.10-0.69]** | **0.22 [0.16-0.35]** | **N.A.** | **N.A.** | **0.06** | **0.22** |
| **Total Conj.**  **at Peak #2**  (mole fract.) | **N.A.** | **0.32 [0.15-0.50]** | **0.86 [0.55-1.00]** | **0.84 [0.58-1.00]** | **0.86 [0.55-0.97]** | **N.A.** | **N.A.** | **0.034** | **0.62** |
| **Total bile salts**  **at Peak#2**  (μmol/L) | **N.A.** | **88.8 [27.3-150.2]** | **361.5 [12.8-1605.9]** | **605.6 [12.8-1605.9]** | **361.5 [124.6-1048.5]** | **N.A.** | **N.A.** | **0.24** | **1.0** |

Data are shown as median and range [minimum to maximum value]. Statistical significance was accepted at P<0.05 corrected for the number of comparisons made (post-hoc Bonferroni-Holm correction) and is demarcated by * in the table.

HC, healthy controls; PBC, primary biliary cholangitis; PSC, primary sclerosing cholangitis; MRS, Mayo Risk Score; TBS, total bile salts; FGF19, Fibroblast Growth Factor 19; C4, 7α-hydroxy-4-cholesten-3-one; CDCA, chenodeoxycholic acid; UDCA, ursodeoxycholic acid; Unconj., unconjugated; Conj., conjugated; Prim., primary; Sec., secondary.
